# Author index

**DOI:** 10.1038/sj.bjc.6690639

**Published:** 1999-08


					Aalame N 693
Aaltomaa S 477, 2001
Aass N 249
Abe H 236
Abe T 556, 1115, 2034
Abels C 360
Aebi S 699
Aeppli DM 1175
Agostino M 964
Ah-See AK 262
Ahlman H 1259
Ahlner-Elmqvist M 766
Ahrens K 73
Airola K 733
Akagi Y 1506
Akaike T 1945
Akao Y 914
Akbulut H 1630
Akgul H 1630
Al-Saffar NM 1035
Ala-Opas M 477, 2001
Alasio L 519
Albrecht C 801
Alexander CL 1494
Ali S 1575
Alitalo K 309
Allal AS 1588
Allalunis-Turner MJ 104
Allgayer H 1884
Alvarez-Mon M 229
Amelotti M 331
Ammon HPT 756
Amoh Y 194
Amos CI 453
An H-X 1920
Andersen A-MN 609
Andersen LJ 412
Andersen PK 609
Anderson H 219
Ando Y 1982
Andrew AC 1803
Andrieu N 1042
Appleby PN 930
Arends J-W 1214
Armstrong B 563
Armstrong RA 1026
Asakura T 711
Asselain B 536
Assiri A 1265
Atkin SL 898
Auerbach L 874
Aurias A 1042
Ausch C 874
Aveyard JS 904
Ayres M 1405
Ayyildiz A 1630
Bacher T 1582
Baggett B 1005
Baguley BC 716
Bahlitzanakis N 1792
Bailey D 569
Balkwill FR 315, 1236
Balonov M 1461
Banerjee D 569
Bang Y-J 1144
Bani MR 504
Banks RE 273
Bareck E 1797
Baretton GB 546
Barkardottir RB 879
Barnetson RStC 1501
Barni S 1595
Barth S 1214
Bartlett JMS 685
Bartoli C 519
Bartus RT 964
Barzan L 614
Barzilay G 940
Basolo F 579
Bastit P 221
Bataille V 295
Batchelor D 1380
Batlle AM del C 1525
Bauer HC 1809
Bauer P 1987
Baumal R 569
Baumler W 360
Beale P 1786
Bebb DG 1979
Beckmann MW 879, 1920
Becroft DMO 1483
Bedane C 1767
Begent R 922
Begg A 1635
Begleiter A 1223
Beijnen JH 1380
Belinsky MG 1342
Bell DR 396
Bender HG 1920
Benedetti Panici P 1665
Benitez J 879
Bennett DC 295
Bennett WP 1445
Benoy I 892
Benraad ThJ 286
Beppu T 1945
Berchuck A 1575
Bergaglio M 1114 (L)
Bergamaschi D 971
Bergenheim AT 142
Berger P 1582
Berger U 419
Bergman AM 981
Bergman J 513
Bergstrom C 1635
Bergstrom JD 650
Bernstein L 1852
Beroukas K 1792
Berrisford C 219
Bertelli G 1114 (L)
Besser GM 1815
Beusenberg FD 1380
Bevilacqua G 11
Bhujwalla ZM 1005
Bicknell J 44
Bieche I 879
Bignon Y-J 879
Bilous M 563
Biorklund A 766
Birch JM 9
Birembaut P 1306
Birnbaum D 879
Bishop DT 295
Bishop GA 1501
Bjordal K 766
Bjork-Eriksson T 1400
Bjorkholm M 1156
Blanchard D 1995
Blanco J 1169
Bleehen NM 1786
Bloigu R 879
Blomqvist C 167, 1763
Bobrow LG 1608
Bock M 67 (SC)
Bodis S 693
Bokemeyer C 801
Boll R 175
Bolt-De Vries J 286
Bonfanti M 971
Bonilla F 1262
Bonora MG 519
Booth C 1550
Borg A 879
Borresen-Dale A-L 879
Bory J-P 1306
Bosomworth M 273
Bourgarel V 1162
Bowman KJ 1738
Boxer G 922
Boyd D 1412, 1884
Boyd RS 315
Boyer MJ 242
Boyle KP 1803
Boysen M 766
Braguer D 1162
Brasseur N 1533
Bratilova AA 1461
Bratina G 403
Braun A 1987
Braun RD 117
Breast Cancer Somatic Genetics
Consortium 879
Bressac-de-Paillerets B 1042
Breton P 87
Briand C 1162
Brodowicz T 1350
Broet P 536
Broggini M 338, 991
Broitman SA 1132
Broni J 1565
Brotherick I 1062, 1271
Brown CL 1012
Brown EH 1525
Brown JM 1245
Brown LM 1830
Brown R 242, 338
Browne S 2025
Browne WL 716
Brunner N 495
Bubley GJ 699
Budinsky A 1350
Bugat R 364
Bugdayci R 1303
Buglova E 1461
Buick RN 1123
Bundred NJ 1265
Burger MPM 38
Burke F 1236
Burkhart CA 1020
Burns PA 1803
Burt EC 9
Butkiewicz D 1445
Butler AR 1525
Butow PN 242, 427
Buxton EJ 1803
Buzard G 458
Caldas C 1968
Caligo MA 11
Callahan R 821
Calvert AH 1738
Calvo S 579
Camacho FI 1427
Camaris C 563
Campani D 579
Campbell F 834
Campbell FC 1062
Campbell IG 70
Campbell NC 1275
Canal P 364
Candel MT 786
Cano J-P 1726
Cao Z 716
Cappelletti V 1150
Carbone DP 483
Carles G 1162
Carpenter CL 1852
Casas A 1525
Cascinu S 1595
Caserini C 1912
Cassagnau E 1995
Cassidy J 862, 1275
Castelli G 1103 (SC)
Catalano G 1595
Catapano CV 971
Author index
Key to abbreviations:
(BR) Book reviews; (L) Letters to the editors; (SC) Short communications
2040
British Journal of Cancer (1999) 80(12), 2040-2047
(c) 1999 Cancer Research Campaign
Article no. bjoc.1999.0639
Cavalli F 1058
Cecchetti D 579
Cellerino R 1595
Cenni B 699
Cerhan JR 1476
Chacon I 1427
Champeme M-H 879
Chan KK 1492
Chang K-J 1838
Chang Y-C 206
Chaplin DJ 117, 724
Charles MW 627 (L)
Charlett A 219
Chatelut E 364
Chattoraj SC 1132
Chen C-H 468
Chen C-J 598
Chen D-S 468
Chen G-D 483
Chen J 1979
Chen J-Y 483
Chen N 946
Chermann J-C 87
Chern H-D 598, 1838
Chessells JM 909
Chevreau P 364
Chi C-W 483
Chiari S 403
Chiarotto JA 1518
Chilvers CED 1859
Ching L-M 716
Chisholm M 279
Chiu BC-H 1476
Chiu Y-H 598
Cho E 1682
Chorazy M 1445
Chou H-C 468
Chow K-C 1617
Christen RD 699
Christensen IJ 495
Christensen TB 1577, 1582
Christodoulou M 1792
Chuang L-Y 705
Churchman M 70
Ciccarelli G 639
Ciccolini J 1726
Ciro M 991
Clark PI 1392
Clarke F 1958
Clarke PA 1035
Clarke R 1682
Clausen OPF 526
Clavel C 1306
Clayton RN 44
Cleton-Jansen A-M 879
Coffey Jr RJ 1012
Colella G 338
Coleman R 221
Coley H 1190
Collins S 1958
Constandinou C 604
Conti A 1058
Conti E 614
Cook PA 1392
Cooke BT 1501
Cooper CS 1565
Cooperative Group of
Docetaxel for Pancreatic
Cancer in Japan 438
Corbacho C 1262
Cortes-Funes H 229
Costa-Pereira AP 371
Cotter TG 371
Coulson JM 1935
Coupland CAC 1859
Cozzi P 991
CRC Phase I/II Committee 1786
Crocker J 604
Crompton MR 1236
Croquette M-F 1042
Crowther D 1405
Cruz MA 1427
Cullen MH 1392
Cummings J 1026
Cunningham D 269
Cunningham DC 1035
Cuq P 1726
Curphey TJ 1223
Curtin NJ 1738
Cuzick J 295
Cvetskovska E 1400
Czyzewski K 1445
Dahia PLM 383
Dallegri F 331
Damstrup L 1012
Danesh J 927
Danielsen T 1697
Dapino P 331
Date M 194
Daver A 536
Davey G 1859
Davey GK 1470
Davidson K 1332
Day NE 1968
De Angelis PM 526
de Bono J 1786
de Both NJ 1652
De Campos ES 396
de Castellarnau C 1169
de Jong J 1058
de Rooij MJM 883
De Stavola BL 1098
De Stefani E 591
de Takats P 1864 (BR)
de Vries EGE 629
de Vries TJ 883
de Wilt JHW 161
de Wit R 1387
de Witte JH 286
Dean R 964
Deans B 940
Debre M 1042
Degiannis E 32
Deichmann M 67 (SC)
Del Mastro P 1665
Delaunay M 1767
Delcros J-G 1512
Delfino L 639
Demidchik EP 1461
Demirci S 1630
Dempke W 1955
Dempsey PJ 1012
Denda K 301
Denekamp J 724, 1635
Deneo-Pellegrini H 591
Denis M 1995
Denis MG 1995
Dennis I 1786
Depisch D 1797
Dettmar P 419
Devilee P 879
Dewhirst MW 117
Dey P 1958
Di Felice G 407
Di Fronzo G 1150
Diaconu C-C 1197
Diaz de Stahl T 650
Diebold J 546
Diehl V 1214
Dillon SA 1046
D'Incalci M 338, 971, 991
Dinjens WNM 1652
Dirix LY 892
DiStefano F 1035
Dockerty JD 1483
Doherty GP 1223
Doi K 1945
Dolan K 834
Dombernowsky P 412
Dominguez G 1262
Donadio M 821
Dorant E 1107
Dosaka-Akita H 1289
Dotzlaw H 379
Doyle E 1380
Droese M 73
Drozdovitch V 1461
Dublin EA 1608
Dufmats M 843
Dumontet C 1162
Dunn TJ 117
Dutton A 604
Dyer MJS 1491, 1565
Eardley A 444
Earle CC 815
Eastell R 1265
Eaton NE 930
Edstrom S 1400
Edward M 1494
Edwards SM 1565
Eeles R 1565
Eggermont AMM 161
Eisenhauer EA 629
Eisinger B 1440
Elamin F 79
Elder RH 940
Elhan HA 1630
Ellis IO 352
Ellis LM 1506
Ellison DW 1322
Elwood JM 1483
Emanuels AG 38
Emerich DF 964
Eng C 383
Engbaek F 1577, 1582
Engeland K 1069
Engert A 1214
Enomoto T 458
Erba E 971
Ercoli A 1665
Eremin O 262
Eroglu A 1630
Eskelinen M 477, 2001
Espana P 1262
Esquivel F 229
Evans D 604
Evans JW 1245
Evans TRJ 1
Evans WK 815
Evensen J 766
Everhart J 1830
Evrard A 1726
Ewers S-B 396
Faggioni A 1103 (SC)
Faircloth G 971
Fairlie DP 1252
Fait E 724
Fajardo I 17
Faretta M 971
Farrell N 1912
Farrell WE 44
Farthing MJG 322
Favero A 614
Ferno M 1453
Ferrandina G 1665
Ferrara GB 639
Field WN 1734
Findlay E 1844
Fink D 699
Fioravanti L 1150
Firusian N 1955
Fischer K 1204
Flaitz CM 1412
Flavell K 604
Flavin A 1786
Flori AR 1312
Flynn A 922
Foekens JA 286
Fontanini G 579
Ford J 221
Forman D 1859
Fornaciari G 11
Forrer M 185
Fossa SD 249, 1392
Foulkes WD 70
Fourkour A 883
Fraioli R 639
Franceschi S 614
Francis JL 279
Franko AJ 104
Frau A 786
Fraumeni Jr JF 1830
Frenander J 1453
Fretigny E 364
Froberg MK 1175
Frojdman K 149
Frontini L 1595
Frost V 670
Froudarakis M 1792
Fujii S 1658, 1945
Fujii Y 1982
Fujime M 301
Fujimoto J 827
Fujise Y 133
Fujita K 301
Fujita M 458
Fukumoto M 775
Fukushige S 556
Fumagalli L 407
Funovics J 1797
Furihata M 256
Furuse K 1435
Fusco V 1595
Futreal PA 1575
Gabriel R 1306
Gaken J 79
Author index 2041
British Journal of Cancer (1999) 80(12), 2040-2047
(c) 1999 Cancer Research Campaign
Gallinari F 991
Gapstur SM 1476
Garambois V 1373
Garcia JF 1427
Garcia JM 1262
Garcia-Castro I 229
Gardner C 1786
Gatti RA 1979
Geirsdottir EK 879
Gelmon K 1979
Gentile G 1103 (SC)
Gentile M 843
Geoffrois L 1767
George MD 87
Georgoulias V 1792
Geradts J 1175
Gerl A 1392
Geroni C 991
Ghielmini M 1058
Ghimenti C 11
Giacomini P 639
Giatromanolaki A 1792
Gibson BE 909
Gibson SL 998
Gill S 1565
Gillies RJ 1005
Gimm O 383
Giorda E 639
Giordani P 1595
Gissler M 1092
Giuliani FC 1912
Giulianotti PG 11
Giuliodori L 1595
Glanzmann C 693
Glaser T 756
Gleave EN 1296
Glickman BW 1979
Goedkoop R 786
Goepfert H 1412
Gokhale DA 1405
Goldbohm RA 1107
Golding BT 1738
Goldstein A 1830
Goldstein AM 295
Golovneva A 1461
Goncalves A 1162
Gonzalez R 1262
Gorostidi B 786
Gorter A 1718
Gossling A 383
Goulko G 1461
Graeff H 419
Grebenschikov N 286
Green A 922
Greenberg RS 1830
Greyling D 32
Griffin RJ 1738
Griffin SM 1271
Griffiths JR 1035
Griscelli C 1042
Groenfeld LE 489
Groopman JD 59
Groscurth P 756
Gruber A 1156
Grunt TW 1350
Gruppo Italiano Malattie
Ematologiche dell' Adulto
(GIMEMA) 1103 (SC)
Gudmundsson J 879
Guichard S 364
Guillou L 185
Gullu I
*
1865 (L)
Gumbs C 1575
Gunn J 2019
Gustavino C 1114 (L)
Ha A 1075
Haaparanta M 513
Haba T 458
Habuchi T 904
Hadfield JA 1550
Hagiwara A 2034
Haier J 1867
Haites NE 862
Hakozaki M 1420
Halliday GM 1501
Halme M 1781
Hamilton SR 59
Hammerlid E 766
Hansen C 1252
Hansen H 1214
Hara T 1898
Hara Y 1982
Harada H 1281
Harasim J 1445
Harbeck N 419
Hargreaves DF 1550
Harland M 295
Harley V 149
Harn H-J 206
Harnden P 273
Harper P 269
Harris CC 1445
Harris M 1405
Harris SR 1905
Hartmann H 73
Hartmann JT 801
Hasan T 344, 946
Hashidume Y 711
Hashimoto T 1623
Hasina R 1708
Hasler B 964
Haslett C 1026
Hassinen I 25
Hatamochi A 1087
Hatano K 1927
Hatori M 954
Hatzitheofilou C 32
Hauss J 1069
Havens JJ 998
Havsteen H 412
Hawkey CJ 1012
Hayashi H 775
Hayashi M 1623
Hayashi S 1623
Hazama S 1420
Hazel DL 918
He X 1005
Healy JC 808
Hebeda KM 744
Heidenreich WF 1461
Hejna M 1350, 1797
Heldin N-E 650
Hellan M 874
Hemminki E 1092
Hendry JH 940
Hennebelle I 364
Henriksson R 142
Herben VMM 1380
Herbison GP 1483
Hermansson A 650
Herring CJ 940
Herrstedt J 412
Hescheler J 1204
Heuvel JJTM 286
Heys SD 262
Hiai H 855
Hickey KP 1803
Hickish T 269
Hicks MJ 1412
Hickson ID 940
Higashi T 1820
Hilakivi-Clarke L 1682
Hilf R 998
Hill A 269
Hill RP 1518
Hill SA 724
Hino S 448
Hinrichs U 1359
Hirai Y 621
Hirano T 1435
Hiraoka M 387
Hirata I 914
Hirose K 1420
Hirose R 827
Hocking DJ 1245
Hofler H 419
Hollema H 38
Holten-Andersen MN 495
Hommura F 1289
Hong RL 1962
Honma Y 96
Honn SE 453
Hoogenboom HR 1214
Hoover RN 1830
Hope T 792
Horii A 556
Hornedo J 229
Horwich A 1392
Horwitz SB 1020
Hospers GAP 629
Houlston RS 1569
Howell A 1265
Howell SB 699
Hoyer-Hansen G 495
Hsu C-H 1080
Hsu MM 1962
Hsu S-M 1838
Hu Y-X 1075
Huang B 1738
Huang C-S 1838
Huang G-T 468
Huang S-Y 468
Huget P 892
Hugh TJ 1046
Humphrey G 918
Hung W-C 705
Hurren R 1123
Huson SM 626 (BR)
Hussey HJ 1231
Hutcheon AW 262
Hutchison C 1786
Huusko P 879
Ichigo S 827
Iglehart JD 1575
Iglesias J 569
Iizuka N 1420
Ikebe N 1945
Ikegami M 914
Imada H 236
Imai K 1623
Imai S 775
Imai T 1648
Imamura M 1281, 1366
Inazawa J 2034
Inoguchi E 1420
Inokuchi H 194
Inoue K 194
Inoue M 458
Irimura T 301
Isemura M 1115
Ishii E 1898
Ishikawa T 256
Ishikawa Y 1623
Ishizaki M 1820
Ito A 1137
Ito D 954
Ito Y 1982
Iwai M 1137
Iwase H 1982
Iwata H 1982
Izbicka E 1332
Jackel A 67 (SC)
Jackson TL 1747
Jacob P 1461
Jacobs E 352
Jacobs I 1826 (SC)
Jacobs IJ 1644
Jacobsen KD 249
Jahkola T 167
James LA 9
Janicke F 419
Janin N 1042
Jannert M 766
Jawhari AU 322
Jayson GC 444
Jazayeri J 657
Je
 drus S 1599
Jefferies S 1565
Jenkins PJ 1815
Jepps HM 1803
Jernstrom H 1453
Jeyarajah A 1826 (SC)
Jeyarajah AR 1644
Jiang S-Y 206
Jichlinski P 185
Jimeno J 971
Jockel K-H 1440
Joensuu H 1763
Joffe JK 269
Johanson V 1259
Johansson M 142
Johnson JG 676
Johnson NW 79
Jones WG 1392
Jong H-S 1144
Joseph WR 716
Jouan H 1512
Judson I 1786
Juszczak E 1844
Kaasa S 766
Kaatsch P 585
Kahlos K 25
2042 Author index
British Journal of Cancer (1999) 80(12), 2040-2047 (c) 1999 Cancer Research Campaign
Kaletsch U 585
Kamby C 1577
Kamijo R 954
Kamps JAAM 1718
Kanatani Y 96
Kanazawa K 621
Kanda S 309
Kandioler-Eckersberger D
1350
Kanetake H 309
Kang SH 1144
Kano M 1281
Karat D 1271
Kariniemi A-L 733
Karlsson E 1400
Karonen T 733
Karrer S 360
Kashu I 387
Katagiri A 1648
Katalinic A 1069
Kates R 419
Kato D 946
Kato H 1435
Kato I 1708
Katoh H 1289
Katsu K 914
Katz F 909
Kavallaris M 1020
Kawabata K 775
Kawakami Y 1289, 1623
Kawamata H 448
Kawana T 621
Kaye SB 1, 1392
Kelso MJ 1252
Kemp B 453
Kenigsberg Y 1461
Kennedy JC 676
Kennel SJ 175
Kerbel RS 504
Kergozien S 1512
Keri G 1197
Kerkhofs L 1052
Kerr DE 1747
Keski-Oja J 733
Key TJ 930, 1470
Kikuchi T 1115
Kilic B 1303
Kim BS 964
Kim H 850
Kim H-K 699
Kim JJ 850
Kim NK 1144
Kim S-J 1144
Kimura M 1648
Kingston JE 808
Kinnula VL 25
Kinoshita I 1289
Kinoshita Y 236
Kinsella AR 1046
Kinugasa N 1820
Kinukawa N 1898
Kitayama J 1927
Kjaer M 412
Kjaerbol A-G 412
Klapthor B 724
Klein J-LD 87
Klenk U 546
Klijn JGM 286
Klimka A 1214
Knowles MA 904
Knuuti J 513
Knuuttila A 1781
Ko JY 1962
Kobayashi S 1982
Kobayashi Y 1623, 1820
Kocaoglu H 1630
Kohler JA 1322
Kohshi K 236
Koji T 309
Kok TC 1052
Koller-Lucae SKM 1542
Kollmannsberger C 801
Kol
osza Z 1599
Konaka C 1435
Konerding MA 724
Konietzko N 1987
Koning GA 1718
Konopacka M 1599
Kornek GV 1797
Kosma V-M 477, 2001
Kostantelos J 1792
Koudstaal J 38
Koukourakis MI 1792
Koumiotaki S 1792
Kovacs G 1565
Kovacs MS 1245
Kovats E 1797
Koyama K 2034
Krammer PH 1689
Kratzke RA 1175
Kricker A 563
Krtolica A 1875
Krucher NA 1875
Kruh GD 1342
Kruit WH 1387
Kruk J 1461
Krzyzowska-Gruca S 1445
Kubista E 874
Kubota Y 194
Kucera P 185
Kuczyk MA 801
Kuiper CM 981
Kunugita N 236
Kuopio T 149
Kurachi H 458
Kurihara M 438
Kurtz JM 1588
Kurtzberg J 87
Kuwada SK 1012
Kvinnsland S 1681
Kyrias G 1792
Kyrodimou E 44
La Madeleine C 1533
La Vecchia C 614
Laake K 879
Labianca R 1595
Lacombe D 221
Laine H 513
Lambers AR 1575
Landthaler M 360
Lang F 1797
Langdon SP 685
Lange N 185
Langer SW 412
Larsson O 1809
Lauge A 1042
Launonen V 879
Laurence R 1332
Laurencet F 1588
Law J 569
Lawson JS 1678 (L)
Lazo PA 2008
Leach MO 1035
Leahy M 269
Leconte A 1373
Lee H-S 468
Lee MM-S 206
Lee P-H 468
Leedman PJ 657
Lehti K 733
Lei M 569
Leirdal M 1558
Leith MK 1223
Lemke H 1214
Leonard R 221
Lesimple T 1767
Levy RD 32
Lewis I 918
Lewis ME 1483
Leygue E 379
Li G 676
Li M 1123
Liaw Y-F 598
Lidereau R 879
Lien HH 249
Lillemoe KD 1830
Lin J-T 468
Lin S-R 1080
Lin Y 504
Lin Y-H 468
Lin Y-W 468
Lin Y-Y 483
Lindblom A 11
Ling V 1123
Linnainmaa K 25
Lipponen P 477, 2001
Liscia DS 821
Lissoni A 403
Lissoni P 407
Lister TA 1815
Little J 1275
Little MP 627 (L)
Liu SS 1828 (SC)
Liu T 11
Liu VWS 1828 (SC)
Liu W 1506
Lizon J 786
Lochon I 364
Logmans A 1387
Lohi J 733
Lohrs U 546
Loirat MJ 1995
Looijenga LHJ 1571
Look MP 286
Lorenzato M 1306
Lorigan P 2025
Loughlin PJ 1738
Lowe D 604
Lower R 1312
Lowes MA 1501
Lu B-X 59
Lu F-J 468
Lu L-M 855
Lubecka B 1599
Lubkin SR 1747
Ludlow JW 1875
Lund H 412
Lundin J 1763
Luporini G 1595
Lustenberger P 1995
Lutz CT 1476
Lwaleed BA 279
Lyons JC 1892
McCall JL 2019
McCluggage WG 70
McConnell D 427
McCoy CL 1035
MacDonald N 1826 (SC)
MacDonald ND 1644
MacDougall JR 504
McGown AT 1550
McGown G 9
McGurk M 1296
Mach JP 1373
Macham MA 1786
Mackean M 269
MacKie RM 1494
McKinney PA 1844
MacKinnon AC 1026
MacKinnon J 940
MacLennan K 918
McLeod HL 862
MacNeil S 2025
Maconochie NES 1098
MacRobert A 1525
Maeda H 1945
Maemura K 914
Maestroni G 1058
Maggiano N 1665
Magnay JL 44
Magnusson B 1400
Maguire P 1770
Mahoney B 1005
Mairs RJ 909
Maitland NJ 1565
Makino T 1281, 1366
Malkusch W 724
Malusecka E 1445
Mancini M 331
Mancuso S 1665
Mandelli F 1103 (SC)
Mangeonjean C 1306
Mangioni C 403
Mangold G 1332
Mangtani P 1098
Mansi J 269
Mantyla M 1781
Manusama ER 161
Manzotti C 1912
Marangoz S 1865 (L)
Marchini S 338, 991
Margison GP 940
Marijnissen JPA 744
Marks A 569
Marks JR 1575
Marone M 1665
Marqversen J 1577, 1582
Marsh DJ 383
Martayan A 639
Marti A 185
Martin-Satue M 1169
Martinez-Montero JC 1427
Martino P 1103 (SC)
Masson D 1995
Author index 2043
British Journal of Cancer (1999) 80(12), 2040-2047
(c) 1999 Cancer Research Campaign
Massuti B 786
Masure M 1306
Matsui E 1435
Matsumoto A 914
Matsumoto K 1708
Matsumoto M 256
Matsumoto T 301
Matsumoto-Taniura N 1708
Matsunaga M 556
Matsuno S 438
Matsushita M 194
Matsuzaki A 1898
Matsuzaki K 194
Matsuzawa E 133
Matthey B 1214
Mattson K 1781
Mayer A 563
Mayer T 1672
Mead GM 1392
Medina MA 17
Mehta R 1412
Meinert R 585
Melbye M 609
Mele A 1103 (SC)
Mendilaharsu M 591
Mengo S 407
Menon U 1644, 1826 (SC)
Mensink EJBM 883
Mercke C 1400
Meregalli S 407
Merrie AEH 2019
Messmann H 360
Metz KA 1440
Mezzelani A 519
Michaeli D 352
Michaelis J 585
Michel J 221
Middleton MR 1679
Mignolet F 221
Miholic J 1797
Miller EP 685
Miller ID 262
Miller JG 2025
Miller SJ 792
Miller WR 685
Millis RR 1608
Minn H 513
Miodini P 1150
Mirzadeh S 175
Mishina T 1289
Miura K 556
Miyazaki S 1898
Miyoshi Y 1185
Mizuta J 775
Mohkamsing S 1571
Mohri S 1898
Mollejo M 1427
Momma T 344
Monarca B 1103 (SC)
Monden Y 1435
Morant R 396
Mori Y 556, 1137
Morikawa H 914
Morikawa T 1289
Morizio R 821
Morland BJ 1301 (L)
Morone P 331
Morris AI 834
Mosca F 11
Mossner J 1069
Motoo Y 1075
Moulinoux J-P 1512
Mountjoy KG 716
MPT Collaborators 1565
Mullen P 685
Mulligan LM 383
Munoz E 1427
Murata Y 458
Murphy G 495
Murphy LC 379
Murray GI 862
Murray JD 1747
Murray P 604
Murrer LHP 744
Muschel RJ 504
Muto T 1927
Mzimba M 273
Nagami H 775
Nagata C 621
Nagawa H 1927
Nagumo M 954
Naher H 67 (SC)
Nakachi K 1623
Nakagawa K 1623
Nakagawa M 775
Nakagawa T 194
Nakagawa Y 914
Nakamura T 1708
Nakamura Y 1185, 2034
Nakashima R 458
Nakashiro K 448
Nakatsukasa H 1820
Narumi K 1115
Nasralla M 1867
Nawa G 1185
Nederbragt H 1359
Negri E 614
Nemechek AJ 1412
Newell DR 1738
Newey C 604
Newlands ES 1392
Newman A 1190
Newton Bishop JA 295
Ngan HYS 1828 (SC)
Nguyen ML 998
Nguyen PL 1175
Nicholson JC 1322
Nicolson GL 1867
Nicolson M 269
Niederacher D 879,
1920
Niehans GA 1175
Nielsen HJ 495
Nielsen SI 1577
Nikaido T 1658
Niki M 914
Nilsson G 1809
Nilsson O 1259
Nio M 775
Nio Y 775
Nishida K 1623
Nishikawa T 914
Nishimura Y 387
Nishiwaki M 133
Nishiwaki Y 133
Nishizawa M 194
Noda K 621
Noda M 322
Noma T 1420
Nomizu M 1898
Norhanom AW 110
Norman A 269
Nortier J 221
Nouso K 1820
Nozawa S 621
Nukiwa T 556, 1115
Nunez de Castro I 17
Nuutinen J 513
Oates J 269
Obwegeser R 874
Ochi T 1185
Ogata N 194
Ogawa M 1945
Ogoshi S 256
Ogston K 262
Ogura Y 1420
Ohashi Y 438
Ohkawa K 711
Ohkawa Y 711
Ohtsubo K 1075
Ohtsubo T 1892
Ohtsuki Y 256
Ojamo M 1459
Oka M 1420
Okada S 438
Okai T 1075
Okamura A 194
Okayasu I 51
Okuno Y 387
Olah E 879
Olbe L 1259
Olden K 87
Oliver RT 1392
Oliver RTD 1859
Olsen K 843
Olsson H 1453
O'Malley Jr BW 1412
Omotehara F 448
Omoto Y 1982
Ono K 387
Onojafe I 1682
Oosterhuis JW 1571
Oosthuizen MMJ 32
Oram D 1826 (SC)
Oram DH 1644
Orii A 1658
Ortel B 946
Osada T 1927
Oshima K 1898
Osin P 1565
Osorio A 879
Ossian K 1042
Ottonello L 331
Ouellet R 1533
Ous S 249
Owczarek S 1599
Pagani O 1058
Paganini-Hill A 1852
Paine-Murrieta G 1005
Palenzona C 821
Palmer BD 716
Palmqvist R 1635
Pampallona S 1058
Panaro NJ 1905
Pancera G 1595
Panda SK 1132
Panzer S 874
Paretzke HG 1461
Park C 850
Park HJ 1892
Parkes SE 1301 (L)
Parkins C 724
Parliament MB 104
Parsons PG 1252
Partridge M 79
Paty PB 1754
Paydas S 1303
Pedersen AN 495
Pelegrin A 1373
Pelliniemi LJ 149
Perego P 1912
Perfekt R 1809
Pessi MA 1595
Peterlin B 879
Peters GJ 981
Peters HA 286
Peterson C 1156
Petridou E 1678 (L)
Petrie K 219
Pezzoni G 1912
Pharoah PDP 1968
Phillips LV 2019
Phillips N 1979
Piao Z 850
Piazza E 1595
Picon AI 1754
Pierotti MA 519
Pignatelli M 322, 1046
Pike MC 1859
Pilotti S 519
Pinedo HM 981
Pink M 964
Pinkerton CR 1190
Piris MA 1427
Pisa P 1156
Pitkanen S 25
Plowman PN 808
Pochtennaja GT 1461
Pogue BW 344
Poikonen P 1763
Polizzi D 1912
Pollanen P 149
Ponder BAJ 295
Popescu N 1905
Porter WH 273
Poston GJ 1046
Potten CS 1550
Potter JD 1476
Pottern LM 1830
Pottier RH 676
Poulsen HS 1012
Pourquier D 1373
Pratesi G 1912
Presek P 1987
Presta M 724
Price A 2025
Pritchard-Jones K 1190
Proebstle TM 1672
Protheroe AS 273
Provencio M 1262
Pruschy M 693
Psarianos T 563
Pukkala E 1459
Pulsoni A 1103 (SC)
Punt CJA 883
Putaud I 1306
Putz V 1204
2044 Author index
British Journal of Cancer (1999) 80(12), 2040-2047 (c) 1999 Cancer Research Campaign
Qian G-S 59
Qiu L 1252
Quereux C 1306
Quesada AR 17
Quillien V 536
Raderer M 1797
Radford JA 444, 1405
Rafferty JA 940
Raghunand N 1005
Ragona G 1103 (SC)
Raimes SA 1271
Rallet A 536
Rao S 269
Raptis L 676
Rashid A 59
Raspaglio G 1665
Ratcliffe WA 1265
Ravaud A 1767
Raygada M 1682
Raymond E 1332
Reeves GK 930
Reimer S 73
Renehan AG 1296
Renshaw J 1190
Resch H 693
Reyes E 229
Reymond MA 1588
Reznek R 1815
Reznek RH 808
Rhemus WE 117
Richardson B 2025
Ricolleau G 536
Riecken E-O 935
Righetti SC 1912
Riley PA 1525
Rilke F 519
Rinaldi F 909
Rio P 879
Ritchie LD 1275
Robert B 1373
Roberts JD 87
Robertson D 1035, 1525
Robertson JFR 352
Rodriguez A 1786
Roe D 1005
Rofstad EK 1697
Rogers E 1265
Rognoni JB 1162
Romain S 536
Roncella M 579
Ronco A 591
Ronen SM 1035
Ronzoni S 971
Rooney PH 862
Roovers RC 1214
Rose C 221
Rosen AC 874
Rosen HR 874
Rosenthal A 1826 (SC)
Rosenthal AN 1644
Rosewicz S 935
Rosing H 1380
Rosner GL 117
Ross FM 1322
Ross RK 1852
Rosso R 396, 1114 (L)
Rota SM 403
Rovelli F 407
Rowan AJ 1569
Rudanko S-L 1459
Rudas M 1350
Ruiter DJ 883
Runnels JM 946
Ruotsalainen U 513
Ruschenburg I 73
Rusin M 1445
Russo I 1682
Rustin GJS 1392
Rutteman GR 1359
Ryder D 1405
Saarialho-Kere UK 733
Saarto T 1763
Saegusa M 51
Saez AI 1427
Safayhi H 756
Sahin B 1303
Sailer E-R 756
St George's Hospital
Collaborators 1565
Saito H 1927
Saito T 1648
Saito Y 309, 1435
Sakaguchi H 827
Sakai H 309
Sakai T 1115, 1137
Sakakura C 2034
Sakata Y 438
Sakuda M 1708
Salgado R 892
Salminen E 149
Salo J 1781
Sanchez B 786
Sanchez E 1427
Sanchez-Beato M 1427
Sanchez-Jimenez F 17
Santella RM 598
Sarkar TK 262
Sarrazin M 1162
Sartor M 79
Sasaki M 458
Sasaki Y 438
Sasazuki T 1884
Sato F 1366
Sato H 1137
Sato M 448, 556, 775
Sato T 1137
Satoh K 1115
Sauer H 1204
Sawabu N 1075
Sawai T 711
Sawamura T 194
Scambia G 1665
Scarffe JH 269
Schabet M 756
Schatzmann E 396
Scheithauer W 1797
Schellens JHM 1380
Scherneck S 879
Scherphof GL 1718
Schiffman M 1830
Schjolberg A 526
Schlott T 73
Schmeller N 546
Schmitt M 419
Schmitz-Drager BJ 1312
Schmoll H-J 801
Schneider J 1987
Schneider SM 1350
Schoenberg JB 1830
Schoengen A 1672
Schott H 1542
Schuller HM 175
Schulz WA 1312
Schuurman AG 1107
Schuz J 585
Schwartz AG 1830
Schwarz MA 1754
Schwarz RE 1754
Schwendener RA 1542
Schwurzer M 1672
Scott D 1271
Scott K 604
Secomb TW 117
Seiki M 1137
Seitz S 879
Seki T 194
Sekiya S 621
Selby P 295
Selby PJ 273
Sellers TA 1476
Selves J 364
Sengupta PS 444
Senter PD 1747
Seo JY 1144
Sessa C 1058
Seth D 657
Sethi T 1026
Setini A 639
Shafford EA 808
Shaheen RM 1506
Sharma S 1332
Shaw K 657
Sheard MA 1689
Sheard T 1770
Sheen TS 1962
Shen C-Y 1838
Shen S-R 206
Shenton BK 1062, 1271
Sheu J-C 468
Shi Y-Q 693
Shibahara S 1945
Shibano K 194
Shields PG 1445
Shiiba K 556
Shimada Y 1281, 1366
Shimizu H 621
Shimo-Oka T 1115
Shinkai H 1087
Shiozaki M 914
Shiozawa A 1658
Shiraishi T 775
Shirasawa S 1884
Shisa H 855
Shousha S 1974
Shyu R-Y 206
Sibley K 1644, 1826 (SC)
Siemers NO 1747
Siersema PD 1052
Sigsgaard T 412
Siim BG 1245
Silva JM 1262
Silva RR 1595
Silvas E 1332
Silverman DT 1830
Simmons S 604
Simon C 1412
Simpson DJ 44
Sinclair AJ 670
Singh PN 273
Sinnett HD 1974
Sioud M 1558
Skaar T 1682
Skarlatos J 1792
Skates SJ 1644
Skegg DCG 1483
Skilleter A 904
Skytting BT 1809
Slade RJ 444
Slevin NJ 1296, 1400
Sludden J 1786
Smith C 563
Smith K 1844
Smith P 1608
Smith PD 1236
Smith PH 273
Smith R 604
Smith TAD 1035
Smyth JF 685, 1026
Snodgrass P 964
Soda H 1332
Sohaib SA 1815
Solovera J 229
Song CW 1892
Southgate J 273
Speirs V 898
Spinelli S 1912
Spitz MR 453
Splinter TAW 1052
Sprangers MAG 1588
Spurr N 879
Spyratos F 536
Stabenow R 1440
Stabin M 175
Stang A 1440
Stanley J 1935
Star WM 744
Stegmaier C 1440
Steingrimsdottir H 79
Stenning SP 1392
Stenwig AE 249
Stephens RW 495
Stevens RG 1459
Stevenson DAJ 862
Steward WP 396
Stierer M 874
Stocker H 396
Stokke T 526
Stone JG 1569
Stoppa-Lyonnet D 1042
Stuart-Smith S 922
Sugase M 621
Sugimori H 621
Sullivan M 766
Sumitani K 954
Sun D 1332
Sunami E 1927
Sunamura M 556
Sundal E 396
Sundstrom J 149
Supino R 1912
Sutherland DR 569
Sutton R 834
Suzuki T 1420
Svensson M 1400
Author index 2045
British Journal of Cancer (1999) 80(12), 2040-2047
(c) 1999 Cancer Research Campaign
Swanson GM 1830
Swanwick MA 1098
Sweep CGJ 286
Sweetenham JW 625 (BR)
Swerdlow AJ 1098
Syrjanen K 2001
Szathmari M 1197
Szeimies R-M 360
Szewczuk MR 676
Sztan M 879
Tagawa K 1087
Taguchi T 438
Takada H 194
Takada M 1435
Takahashi K 1648
Takahashi T 2034
Takeda M 1648
Takemoto H 194
Takenaga K 127
Takizawa K 954
Talaat A 1644
Talamini R 614
Talbot DC 792
Tamaya T 827
Tammilehto L 1781
Tamura K 775
Tanaka M 1115
Tanaka S 1945
Tanigawa N 914
Taniguchi H 2034
Tanikawa T 1648
Tannapfel A 1069
Tannergard P 11
Tanuma J-i 855
Tattersall MHN 242, 427
Tavassoli M 79
Taverna S 971
Taylor BA 1046
Taylor CW 1005
Taylor GM 1405
Teicher BA 699
Telatar M 1979
ten Bokkel Huinink WW 1380
ten Hagen TLM 161
Terashima H 236
Terret C 364
Terskikh A 1373
Tezuka F 1115
Thakker N 79
Thakker RV 44
Thatcher N 219, 396
Thomas HV 1470
Thome M 67 (SC)
Thomson CS 1844
Thomssen C 419
Thorgeirsson UP 1905
Thorncroft M 9
Tilanus HW 1052, 1652
Ting LL 1962
Tisdale MJ 1231, 1734
Tisell LE 1259
Titley J 1035
Todorov PT 1734
Tognella S 1912
Toivonen T 167
Toki T 1658
Tokuchi Y 1623
Tokui N 236
Tomita Y 1648
Tomlinson IPM 70, 1569
Toniolo A 579
Toshikuni N 1820
Tosti ME 1103 (SC)
Toukomaa H 1092
Toulas C 536
Toyama T 1982
Tozer GM 117
Trainer PJ 1815
Trasti H 249
Trichopoulos D 1678 (L)
Tsubono M 775
Tsuchitani T 775
Tsuchiya E 1623
Tsuji T 1820
Tsukifuji R 1087
Tsuno N 1927
Tsurusaki T 309
Tubiana-Hulin M 221
Tucker MA 295
Turner RN 1738
Twelves CJ 1786
Uchida D 448
Uchida M 1115
Uchida S 1281, 1366
Uematsu S 1820
Uemura S 236
Ulm K 419
United Kingdom Testicular
Cancer Study Group 1859
Upton C 1236
Vaalamo M 733
Valencak J 1797
Valenzano M 1114 (L)
Valsuani G 407
van Ackern C 724
van de Locht LTF 883
van Dekken H 1652
van den Bergh H 185
van den Bosch S 883
van den Brandt PA 1107
van der Burg MEL 1387
van der Gaast A 1052
van der Kwast TH 489
van der Saag PT 1571
van der Vijgh WJF 981
van der Zee J 1387
van Gurp RJHLM 1571
van Ijken MGA 161
van Lier JE 1533
Van Marck E 892
van Moorsel CJA 981
van Muijen GNP 883
van Rhoon GC 1387
van Schothorst EM 1571
van Sluis R 1005
van Tiel ST 161
van Tienoven ThH 286
van Tilborg AAG 489
van Zomeren DM 1380
Vansteenkiste JF 396
Varghese G 396
Varley J 879
Varley JM 9
Vasey P 1786
Veerman G 981
Vehmas T 1781
Velikonja N 879
Venesio T 821
Venetianer A 1197
Venturini M 1114 (L)
Verajankorva E 149
Verkasalo PK 1459
Vermeulen PB 892
Vermorken JB 981
Verweij J 1387
Vian L 1726
Villanueva MJ 1262
Villar-Grimalt A 786
Vimala S 110
Vinholes J 221
Virtanen I 167
Visani G 1103 (SC)
Voit C 1672
Volpe R 614
von Deimling A 383
von der Maase H 1577, 1582
Von Hoff DD 1332
von Marschall Z 935
von Smitten K 167
Wada H 458
Waehre H 249
Wagner M 2025
Wagnieres G 185
Wahlberg S 11
Waites A 1635
Wakasa K 458
Waldmann V 67 (SC)
Walker LG 262
Walker MB 262
Walker SJ 834
Wallace RB 1476
Walls J 1265
Walsh GL 453
Wang CC 1962
Wang F 1420
Wang H 1884
Wang J-S 59
Wang J-T 468
Wang J-Y 1080
Wang L-S 1617
Wang X 1223
Wang Z-J 70
Wangberg B 1259
Warnakulasuriya S 79
Wartenberg M 1204
Wasenius V-M 167
Watanabe G 1281, 1366
Watanabe H 1075
Watanabe T 194
Watanabe Y 1435
Waters CM 1026
Waters JS 269
Watson DS 1062
Watson PH 379
Watson SA 352
Weagle GE 676
Webb A 269
Webb JAW 808
Weber L 1672
Wei T 360
Weinans L 1069
Weller M 756
Welsh JA 1445
West CML 1400
West ML 1252
Westergaard T 609
Westin T 766
Weston A 1445
Weytjens R 892
Whelan P 273
Wheldon TE 909
White G 879
Whynes DK 215
Widel M 1599
Widmark A 142
Wiesner B 1987
Wijnhoven BPL 1652
Wildiers J 221
Wiley HS 1012
Wilkinson PM 1392
Williams EMI 834
Williams MV 1392
Williams V 2025
Willis TG 1565
Wilson KE 685
Wilson RG 1062
Wiltschke C 1350
Windle B 1332
Wingren S 843
Winqvist R 879
Winter S 756
Wittekind Ch 1069
Wobbes T 883
Wohlfahrt J 609
Woitowitz H-J 1987
Wojciechowicz DC 1754
Wolf G 874
Woll PJ 1405, 1935
Wolstenholme JL 215
Wouters BG 1245
Wu C-W 483, 1617
Wu HC 344
Wu X 453
Xie K 1506
Xiong H 964
Xu D 1156
Xu W-H 70
Yadav M 110
Yajima A 621
Yamada Y 1898
Yamagishi H 2034
Yamaguchi T 2034
Yamaguchi Y 1075
Yamamoto C 194
Yamamoto M 301
Yamamoto-Yamaguchi Y 96
Yan H-Y 1920
Yan Z-Y 70
Yanagisawa T 1190
Yanagitani S 194
Yang S-A 1080
Yang S-Y 598
Ychou M 1373
Yeger H 569
Yeh M-Y 206
Yiangou C 1974
Yokoyama S 711
Yoshida H 448
Yoshida K 1820
Yoshida N 1898
2046 Author index
British Journal of Cancer (1999) 80(12), 2040-2047 (c) 1999 Cancer Research Campaign
Author index 2047
British Journal of Cancer (1999) 80(12), 2040-2047
(c) 1999 Cancer Research Campaign
Yoshida TO 133
Yoshikawa H 621, 1185
Yoshimura K 1420
Yoshino K 458
Young LS 604
Yu H-S 1080
Yu J-C 206
Yu M-W 598
Yu Z 1979
Yun K 2019
Zaloudik J 1689
Zambon M 219
Zanetta G 403
Zastawny RL 1123
Zborek A 1445
Zebrowski B 1506
Zellweger M 185
Zhai Y-L 1658
Zhang W 453
Zheng W 1476
Zielinski CC 1350
Zompetta C 1103 (SC)
Zunino F 1912
Zvonova I 1461
Zwarthoff EC 489

				
